# Admission Laboratory-Based Prediction of Six-Month Mortality After COVID-19 Hospitalization: Model Development, Bootstrap Internal Validation, and Single-Center Temporal Validation

**DOI:** 10.3390/medicina62081478

**Published:** 2026-07-31

**Authors:** Onur Çelik, Oktay Gülcü

**Affiliations:** 1 Department of Pulmonary Medicine, Erzurum Faculty of Medicine, University of Health Sciences, 25240 Erzurum, Türkiye; 2 Department of Cardiology, Faculty of Medicine, Atatürk University, 25240 Erzurum, Türkiye; droktaygulcu@gmail.com

**Keywords:** COVID-19, six-month all-cause mortality, clinical prediction model, risk stratification, routine laboratory tests, single-center temporal validation, prognostic score

## Abstract

*Background and Objectives*: Early identification of patients at high risk of death after COVID-19 hospitalization may support monitoring, follow-up planning and resource allocation. We aimed to develop and internally validate a parsimonious admission laboratory-based model for six-month all-cause mortality and derive a simplified risk score. *Materials and Methods*: This retrospective single-center cohort included 1827 consecutive adults hospitalized with COVID-19 between 3 March and 6 November 2020. The primary model was developed in 1559 patients with complete predictor data after data-quality review. Prespecified predictors were age, albumin, neutrophil-to-lymphocyte ratio, blood urea nitrogen, lactate dehydrogenase, troponin I and sodium. Model performance was assessed using discrimination and calibration measures, 500-sample bootstrap internal validation and a chronological split-sample assessment in later admissions from the same center; this was internal–temporal rather than external validation. The simplified score was derived exclusively in the chronological development cohort and applied unchanged to the later cohort. *Results*: Among 1827 eligible patients, 437 died within six months (23.9%). The primary complete-case modeling cohort included 1559 patients, of whom 386 died. Older age, lower albumin and higher neutrophil-to-lymphocyte ratio, blood urea nitrogen, lactate dehydrogenase, troponin I and sodium were independently associated with mortality. The model achieved an apparent AUC of 0.936 (95% CI 0.919–0.954) and an optimism-corrected AUC of 0.933. The later same-center cohort yielded an AUC of 0.908 (95% CI 0.871–0.939). The development-derived simplified score had an AUC of 0.927 (95% CI 0.909–0.943) in the development cohort and 0.916 (95% CI 0.885–0.944) when applied unchanged to the later cohort. *Conclusions*: A model combining age with six routinely available admission laboratory variables showed strong discrimination for six-month all-cause mortality after COVID-19 hospitalization and retained good discrimination in single-center temporal assessment. However, calibration drift was observed, and the model and simplified score should currently be considered supportive risk-stratification frameworks rather than deployable stand-alone clinical calculators. External validation, contemporary recalibration and prospective assessment of clinical utility are required before implementation.

## 1. Introduction

Coronavirus disease 2019 (COVID-19), caused by severe acute respiratory syndrome coronavirus 2, has a broad clinical spectrum ranging from mild upper-respiratory symptoms to severe pneumonia, acute respiratory distress syndrome, thromboinflammatory complications, multiorgan dysfunction and death [1,2]. Although respiratory failure is the dominant manifestation in patients requiring hospitalization, clinical outcomes are also influenced by age, comorbidity burden, systemic inflammation and extrapulmonary organ dysfunction [1,2,3,4]. These overlapping pathways make outcome prediction difficult when individual variables are interpreted in isolation and support the use of multivariable approaches that combine complementary domains of risk.

Mortality and morbidity after COVID-19 hospitalization are not confined to the index admission. Patients discharged after severe infection remain at increased risk of readmission, multiorgan dysfunction and death [5]. A six-month outcome therefore provides a broader assessment of prognosis than in-hospital mortality alone and captures both acute deaths and early post-discharge events. From a clinical perspective, identifying patients with sustained medium-term risk at the time of admission may help clinicians plan closer monitoring during hospitalization, anticipate the need for critical-care assessment and recognize patients who may require more structured follow-up after discharge.

Routine admission laboratory tests are attractive for risk prediction because they are rapidly available, inexpensive and widely measured even in resource-constrained settings. Albumin and the neutrophil-to-lymphocyte ratio (NLR) reflect different aspects of the inflammation–nutrition and innate immune response [6,7]; blood urea nitrogen (BUN) incorporates renal, hemodynamic and catabolic stress and has shown prognostic value in hospitalized COVID-19 [8]; lactate dehydrogenase (LDH) reflects tissue injury and has been associated with adverse COVID-19 outcomes [9]; cardiac troponin I identifies myocardial injury [10]; and sodium may reflect disordered fluid balance and illness severity [11]. Age adds a robust measure of baseline vulnerability. A model combining these variables may therefore capture several biologically distinct mechanisms with greater prognostic value than any single marker.

A large number of COVID-19 diagnostic and prognostic models were published during the early pandemic. However, systematic reviews showed that many models were developed using small or selected samples, relied on data-driven variable selection, handled missing data inadequately, reported discrimination without calibration and lacked appropriate internal or external validation [12]. The 4C Mortality Score is a notable exception and demonstrated the value of a structured admission-based tool, but it requires clinical variables such as respiratory rate, oxygen saturation and level of consciousness in addition to laboratory measurements [13]. In many retrospective hospital datasets, these clinical observations are incompletely recorded, whereas routine laboratory variables are more consistently available.

Another important challenge is transportability. Prognostic performance observed in the derivation sample may deteriorate when the model is applied to patients admitted later, even within the same institution, because case mix, admission thresholds, treatment strategies and baseline mortality change over time. Reporting apparent performance alone may therefore overstate clinical reliability. Bootstrap internal validation can quantify model optimism, while a chronological split-sample assessment provides an initial test of temporal stability. Similarly, a simplified integer score can improve bedside interpretability, but it should be evaluated separately because dichotomization and coefficient rounding can reduce information and alter calibration.

In this study, we aimed to develop a parsimonious model based on age and six routinely measured laboratory variables obtained within the first 24 h of hospital admission for predicting six-month all-cause mortality after hospitalization with COVID-19. We sought to assess discrimination and calibration, correct performance for optimism using bootstrap resampling, examine chronological temporal performance in later admissions from the same center, evaluate the influence of extreme sodium values, and translate the model into a clinically interpretable integer score.

## 2. Materials and Methods

### 2.1. Study Design and Population

We conducted a retrospective cohort study at the University of Health Sciences, Erzurum Regional Training and Research Hospital, a tertiary referral center. The source cohort comprised 1827 consecutive adults aged 18 years or older hospitalized with COVID-19 between 3 March and 6 November 2020. COVID-19 was diagnosed by a positive reverse-transcriptase polymerase chain reaction test and/or thoracic computed tomography findings considered compatible with COVID-19 pneumonia in the relevant clinical context. Patients younger than 18 years and records without ascertainable six-month vital status were excluded.

This study was conducted at the same institution as our previously published investigation of platelet large cell ratio as a prognostic marker [14], but it used a distinct patient cohort and addressed a different research question. The previous study evaluated P-LCR as a single hematologic prognostic marker, whereas the present study developed and internally validated a multivariable admission laboratory-based prediction model and simplified score for six-month all-cause mortality.

The study was conducted in accordance with the Declaration of Helsinki and approved by the Clinical Research Ethics Committee of the University of Health Sciences, Erzurum Regional Training and Research Hospital (7 November 2022; approval no. 2022/17-165). The requirement for written informed consent was waived because of the retrospective design.

### 2.2. Outcome

The primary outcome was six-month all-cause mortality after the index hospital admission. Deaths during the index hospitalization were identified from hospital records. Post-discharge vital status was ascertained through e-Nabız, Türkiye’s national electronic health record system, during the six-month follow-up period. In the source database, TOTAL MORT. was coded as 0 for alive, 1 for in-hospital death and 2 for post-discharge or out-of-hospital death within six months; codes 1 and 2 were combined for the primary outcome.

### 2.3. Candidate Predictors and Data Quality

Seven predictors were evaluated: age, serum albumin, NLR, BUN, LDH, troponin I and sodium. These variables were selected a priori on clinical and biological grounds, and because they were routinely available in the source dataset, no automated stepwise or *p*-value-based predictor selection was used. NLR was calculated as the neutrophil count divided by the lymphocyte count. Laboratory predictors were defined as the first available value within 24 h of hospital admission; peak, nadir and whole-hospitalization average values were not used. Cardiac troponin I was routinely requested for all hospitalized patients at admission and was measured using the first available sample obtained within 24 h of hospitalization. A retrievable troponin I result was not available for every patient, and the retrospective records did not reliably distinguish among pre-analytical problems, sample rejection, incomplete testing and electronic-record transfer failures. Serum cardiac troponin I concentrations were measured using a paramagnetic-particle chemiluminescent immunoassay on the UniCel DxI 800 Access Immunoassay System (Beckman Coulter, Inc., Brea, CA, USA). Results were expressed in ng/L, and the local 99th-percentile upper reference limit used for the simplified score was 34 ng/L. Detailed predictor definitions, units, model handling and timing rules are provided in Supplementary Table S2.

During final data verification, the first valid admission sodium results for three patients were confirmed as 160, 166 and 217 mmol/L. To apply a consistent extreme-value quality-control rule and prevent these values from disproportionately influencing the sodium coefficient, values ≥ 160 mmol/L were excluded from the primary modeling cohort. Robustness was assessed in sensitivity analyses that retained all three observations, winsorized values above 160 mmol/L at 160 mmol/L, and modeled sodium categorically (<135, 135–144 and ≥145 mmol/L).

### 2.4. Missing Data

Complete data for all seven predictors were available in 1562 patients; 265 patients were excluded from complete-case modeling because at least one prespecified predictor was unavailable. After the additional data-quality exclusion of three sodium values ≥160 mmol/L, the primary analytic cohort comprised 1559 patients. The primary analysis used complete cases. Complete-case analysis was retained as the prespecified primary framework because the reasons for missing laboratory measurements were not reliably documented, and the plausibility of the missing-at-random assumption underlying the imputation analysis could not be adequately assessed. Multiple imputation was therefore used as a complementary sensitivity analysis rather than as a replacement for the prespecified primary analysis. A 20-imputation chained-equation sensitivity analysis was performed to assess the influence of predictor missingness [15]. The primary cohort derivation is summarized in Table 1 and Supplementary Figure S1.

### 2.5. Statistical Analysis

Continuous variables are presented as mean ± standard deviation or median (interquartile range), as appropriate, and categorical variables as number and percentage. Between-group comparisons used Welch’s *t* test or the Mann–Whitney U test for continuous variables and the chi-square or Fisher exact test for categorical variables. NLR, BUN and LDH were log_2_-transformed; troponin I was transformed as log_2_(1 + x), allowing odds ratios to be interpreted per doubling. Age, albumin and sodium were entered on their original scales. Restricted cubic spline sensitivity analyses using four knots were performed for age and sodium. Likelihood-ratio tests with two degrees of freedom assessed the contribution of the non-linear spline terms relative to the corresponding linear terms. Statistical analyses were performed using IBM SPSS Statistics version 32.0.0.0 (IBM Corp., Armonk, NY, USA) and Python version 3.14.6 (Python Software Foundation, Wilmington, DE, USA).

A multivariable logistic regression model included all seven prespecified predictors without automated selection. Discrimination was quantified by the area under the receiver operating characteristic curve (AUC). The 95% confidence interval for the apparent AUC was calculated using the Hanley–McNeil method [16]. Calibration was assessed by calibration intercept and slope, a LOESS calibration curve with decile points, and the Brier score. A calibration intercept below 0 indicates overprediction of average risk, whereas a calibration slope below 1 indicates predictions that are too extreme [17]. Internal validity was assessed using 500 bootstrap resamples; the entire coefficient-estimation step was repeated in each resample to estimate optimism in discrimination. Sodium-handling sensitivity models retained the full complete-case cohort, winsorized sodium at 160 mmol/L, or replaced continuous sodium with clinically defined categories. Exploratory decision-curve analysis quantified net benefit across threshold probabilities from 0.05 to 0.80 and compared the model with treat-all and treat-none strategies [18].

For chronological temporal validation, patients were ordered according to admission date. The earliest 70% of admissions constituted the development cohort, and the most recent 30% constituted the temporal-validation cohort. The 70%/30% split fell within admissions dated 5 October 2020; records sharing this date were retained in their original source-row order to provide a reproducible tie-breaking rule. The split point was generated by the chronological partition and was not selected on clinical grounds. Model coefficients estimated in the development cohort were applied unchanged to the later cohort. Percentile 95% confidence intervals for temporal AUC were obtained from 500 bootstrap resamples of the later validation cohort. A 20-imputation chained-equation sensitivity analysis assessed the influence of predictor missingness. This was an internal–temporal rather than external validation because both cohorts originated from the same center. The equation reported for the final model was estimated separately in the full primary analytic cohort (*n* = 1559) and was distinct from the coefficient set estimated in the earliest 70% for the chronological assessment. The 4C Mortality Score was not calculated because respiratory rate, oxygen saturation and level of consciousness were not recorded with sufficient completeness and standardization for reliable retrospective scoring.

For bedside simplification, seven clinically interpretable binary thresholds were fixed before score fitting and were not re-estimated in the later cohort: age ≥ 70 years, albumin < 3.5 g/dL, NLR ≥ 10, BUN ≥ 30 mg/dL, LDH > 250 U/L, troponin I > 34 ng/L and sodium ≥ 145 mmol/L. A logistic regression containing these seven binary predictors was fitted exclusively in the chronological development cohort (*n* = 1091; 290 deaths). A transparent three-tier coefficient-magnitude rule converted the development-cohort beta coefficients into one, two or three points; the exact rule and coefficients are reported in Supplementary Methods S6 and Supplementary Table S8. The resulting 0–12 score and risk categories—low (0–2), intermediate (3–5), high (6–8) and very high (≥9)—were fixed before application to the later cohort (*n* = 468; 96 deaths). No threshold, weight or category boundary was modified after examination of later-cohort outcomes. Score discrimination was quantified by AUC with 95% percentile-bootstrap confidence intervals from 5000 resamples, and observed mortality was reported with Wilson 95% confidence intervals in both cohorts. Detailed development and later-cohort results are provided in Supplementary Tables S8 and S9. Reporting followed the updated TRIPOD+AI guidance for clinical prediction models, which also applies to regression-based models [19]. The reporting checklist is provided in Supplementary Table S1; the participant-flow diagram in Supplementary Figure S1; the full model specification and worked example in Supplementary Methods S1; predictor definitions and measurement rules in Supplementary Table S2; chronological partition details in Supplementary Table S3; sodium sensitivity-analysis specifications and summary findings in Supplementary Table S4; the reproducible workflow in Supplementary Methods S2; the multiple-imputation summary in Supplementary Methods S3 and Supplementary Table S5; decision-curve analysis in Supplementary Methods S4, Supplementary Figure S2 and Supplementary Table S6; and restricted cubic spline assessment in Supplementary Methods S5, Supplementary Table S7 and Supplementary Figures S3 and S4.

## 3. Results

### 3.1. Cohort, Outcomes and Missing Data

The source cohort included 1827 patients. Six-month all-cause mortality occurred in 437 patients (23.9%): 377 deaths occurred during the index hospitalization and 60 after discharge or outside the study hospital. Complete data for all seven predictors were available in 1562 patients. After exclusion of three sodium values ≥ 160 mmol/L, the primary analytic cohort comprised 1559 patients (85.3%), among whom 386 (24.8%) died within six months, and 1173 survived.

A total of 268 patients were not included in primary modeling: 265 because at least one predictor was unavailable and three because sodium was ≥160 mmol/L. The primary analytic cohort contained 386 deaths, whereas 51 deaths occurred among patients not included in primary modeling. Cohort derivation is summarized in Table 1.

### 3.2. Baseline Characteristics

Within the primary analytic cohort, non-survivors were older than survivors (74.8 ± 10.9 vs. 62.9 ± 14.7 years; *p* < 0.001) and were more often male (60.4% vs. 50.9%; *p* = 0.001). Hypertension, coronary artery disease, congestive heart failure, COPD and atrial fibrillation were more frequent among non-survivors, whereas chronic kidney disease and diabetes mellitus were not significantly different. Thoracic CT involvement and RT-PCR positivity were similar between outcome groups (Table 2).

### 3.3. Multivariable Model

**Figure 1:**
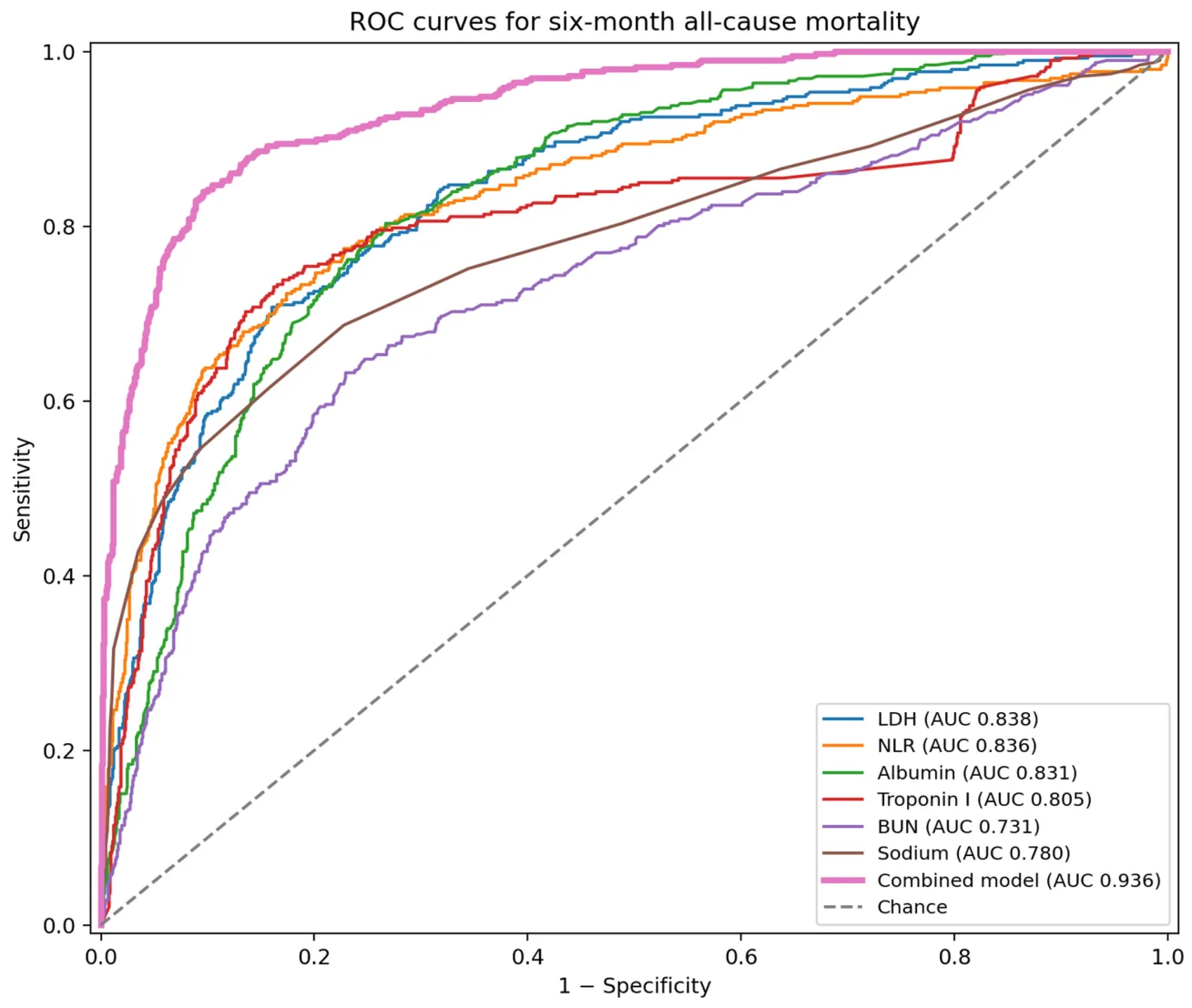
Receiver operating characteristic curves of individual markers and the sodium-outlier-excluded combined model for six-month all-cause mortality.

Receiver operating characteristic curves for the individual markers and the combined model are shown in Figure 1.

**Figure 2:**
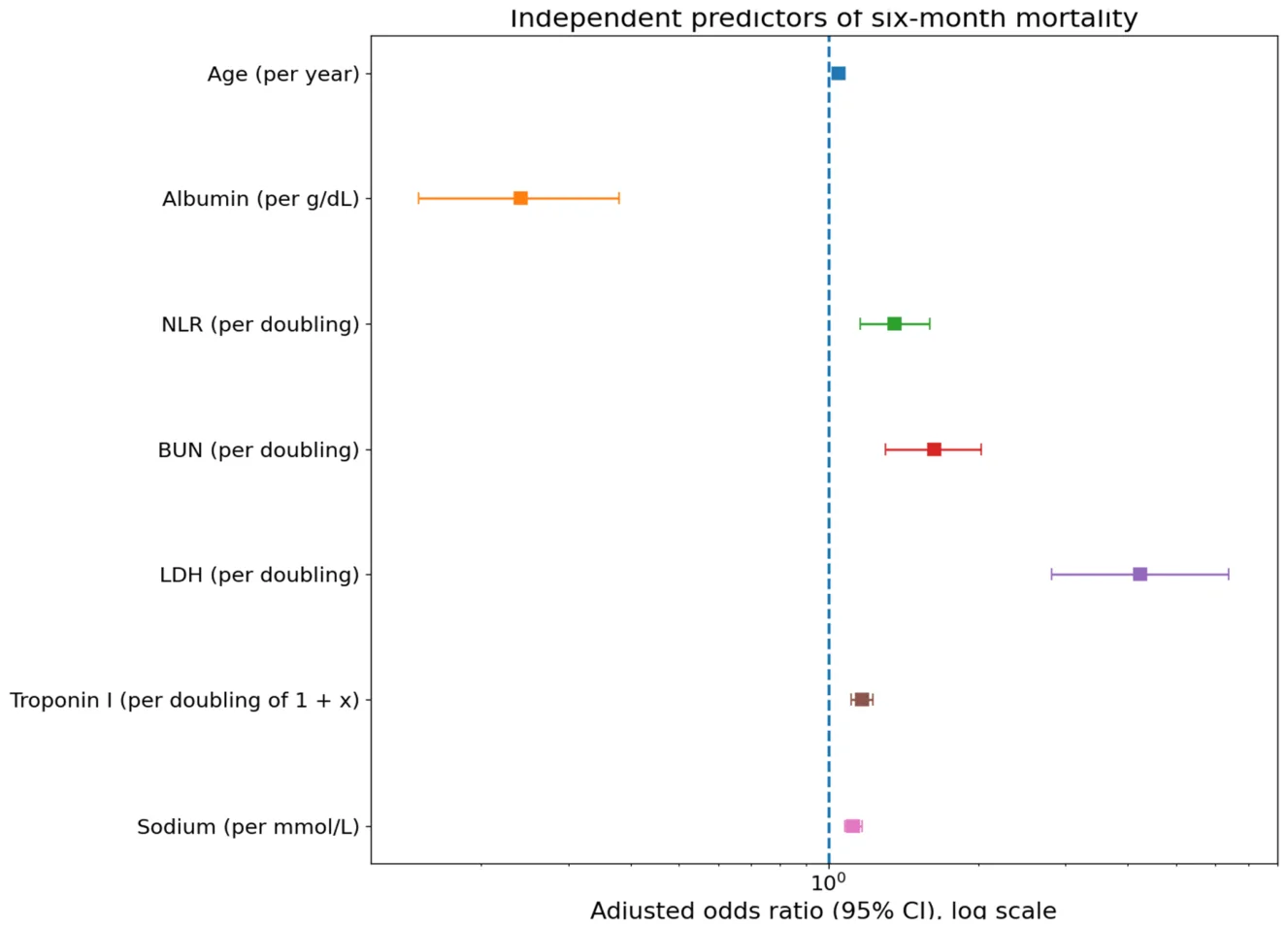
Adjusted odds ratios and 95% confidence intervals in the sodium-outlier-excluded primary model.

All seven predictors remained independently associated with six-month mortality after mutual adjustment. Adjusted odds ratios were 1.05 per year for age, 0.24 per g/dL for albumin, 1.36 per doubling of NLR, 1.63 per doubling of BUN, 4.23 per doubling of LDH, 1.17 per doubling of 1 + troponin I, and 1.12 per mmol/L for sodium (all *p* < 0.001; Table 3 and Figure 2).

The following equation represents the final model estimated in the full primary analytic cohort (*n* = 1559); it was not the coefficient set used to generate the later-cohort temporal assessment: logit(p) = −32.7703 + 0.05021 × age − 1.42211 × albumin + 0.31019 × log_2_(NLR) + 0.48726 × log_2_(BUN) + 1.44255 × log_2_(LDH) + 0.15598 × log_2_(1 + troponin I) + 0.11751 × sodium. The full model specification and a worked probability example are provided in Supplementary Methods S1.

### 3.4. Discrimination, Calibration and Single-Center Temporal Validation

The discriminative performance of the individual markers, including the data-derived Youden cut-offs and associated diagnostic measures, is summarized in Table 4.

**Figure 3:**
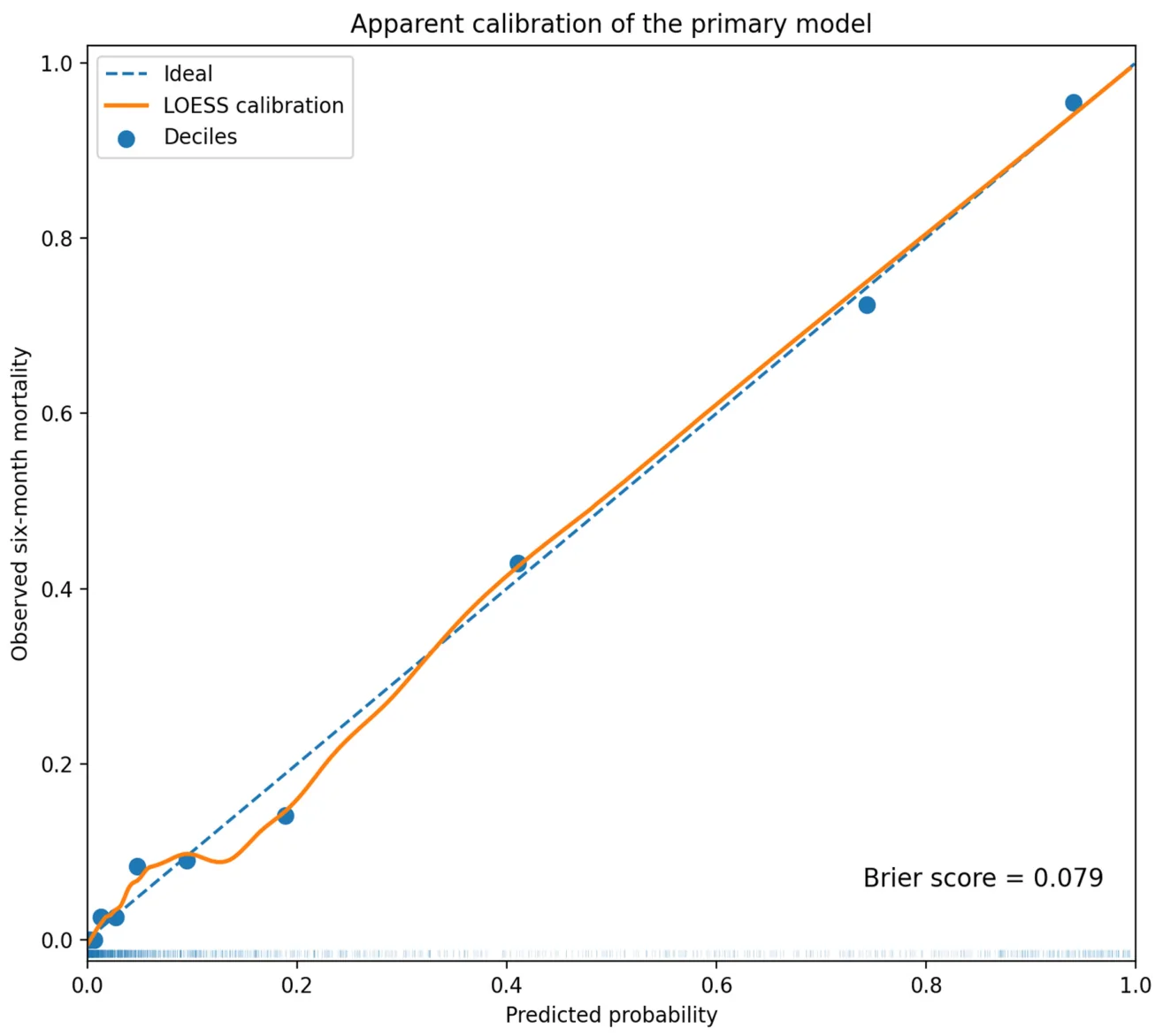
Apparent calibration of the sodium-outlier-excluded primary model with a LOESS curve, decile points and a rug plot.

The primary outlier-excluded model had an apparent AUC of 0.936 (95% CI 0.919–0.954). The optimism-corrected AUC was 0.933, and the apparent Brier score was 0.079 (Table 5 and Figure 3).

**Figure 4:**
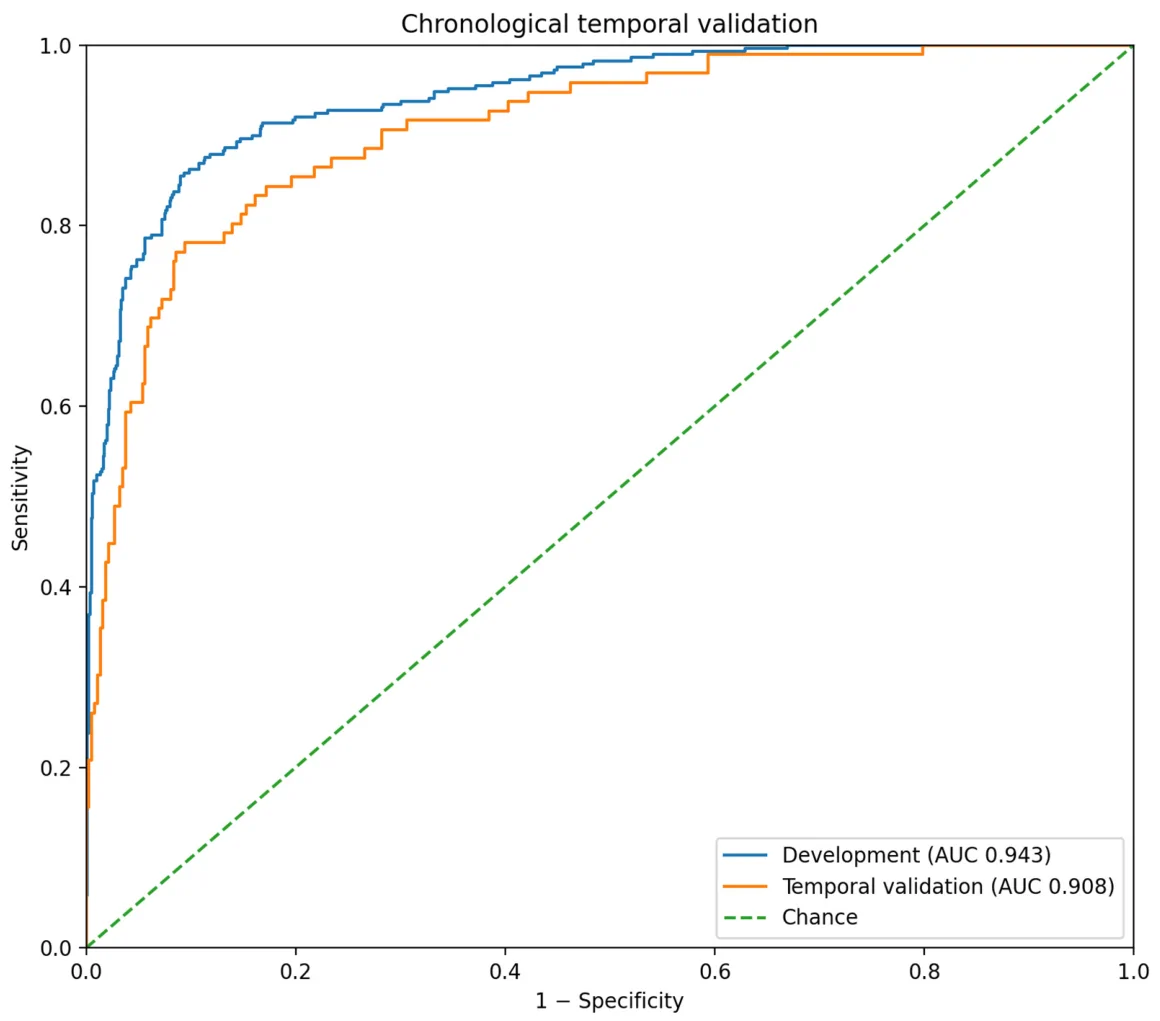
Receiver operating characteristic curves in the chronological development cohort and later same-center temporal-validation cohort after sodium-outlier exclusion.

The reproducible chronological development cohort comprised 1091 patients and 290 deaths; the later validation cohort comprised 468 patients and 96 deaths. In the later cohort, AUC was 0.908 (95% CI 0.871–0.939), calibration slope 0.893, calibration intercept −0.087 and Brier score 0.087 (Table 5 and Figure 4). The slope below one indicated predictions that were somewhat too extreme, while the slightly negative intercept indicated mild overprediction of average risk in later admissions. Thus, although ranking of risk remained strong, absolute-risk probabilities may require recalibration before clinical use. The chronological partition sizes, event rates and later-cohort performance are summarized in Supplementary Table S3. This same-center chronological assessment provides an initial test of temporal transportability, not independent external validation.

Retention of all three sodium values ≥ 160 mmol/L yielded an adjusted sodium OR of 1.09 per mmol/L (95% CI 1.06–1.13) and an AUC of 0.936. Winsorization at 160 mmol/L yielded an adjusted sodium OR of 1.12 per mmol/L (95% CI 1.08–1.16) and an AUC of 0.936, while categorical sodium modeling yielded an AUC of 0.936 and an OR of 9.18 (95% CI 4.38–19.24) for sodium ≥ 145 versus <135 mmol/L. The categorical contrast was specified as part of the sensitivity model and should be interpreted as a robustness analysis rather than as a clinical-threshold effect estimate. Across 20 chained-equation imputations in the sodium-outlier-excluded source cohort (*n* = 1824; 435 deaths), predictor directions were concordant with the complete-case model, and mean MI AUC was 0.929 (range 0.927–0.931). Exploratory restricted cubic spline assessment did not provide evidence of important non-linearity for age (*p* for non-linearity = 0.195), whereas sodium showed modest non-linearity (*p* = 0.015), supporting cautious interpretation of the sodium coefficient and score weight. Exploratory decision-curve analysis showed higher net benefit for the model than treat-all and treat-none strategies across threshold probabilities from 0.05 to 0.80. Full sodium sensitivity results, MI estimates, decision-curve analysis and spline summaries are provided in Supplementary Table S4, Supplementary Methods S3–S5, Supplementary Figure S2, Supplementary Tables S6 and S7.

### 3.5. Simplified Score

**Figure 5:**
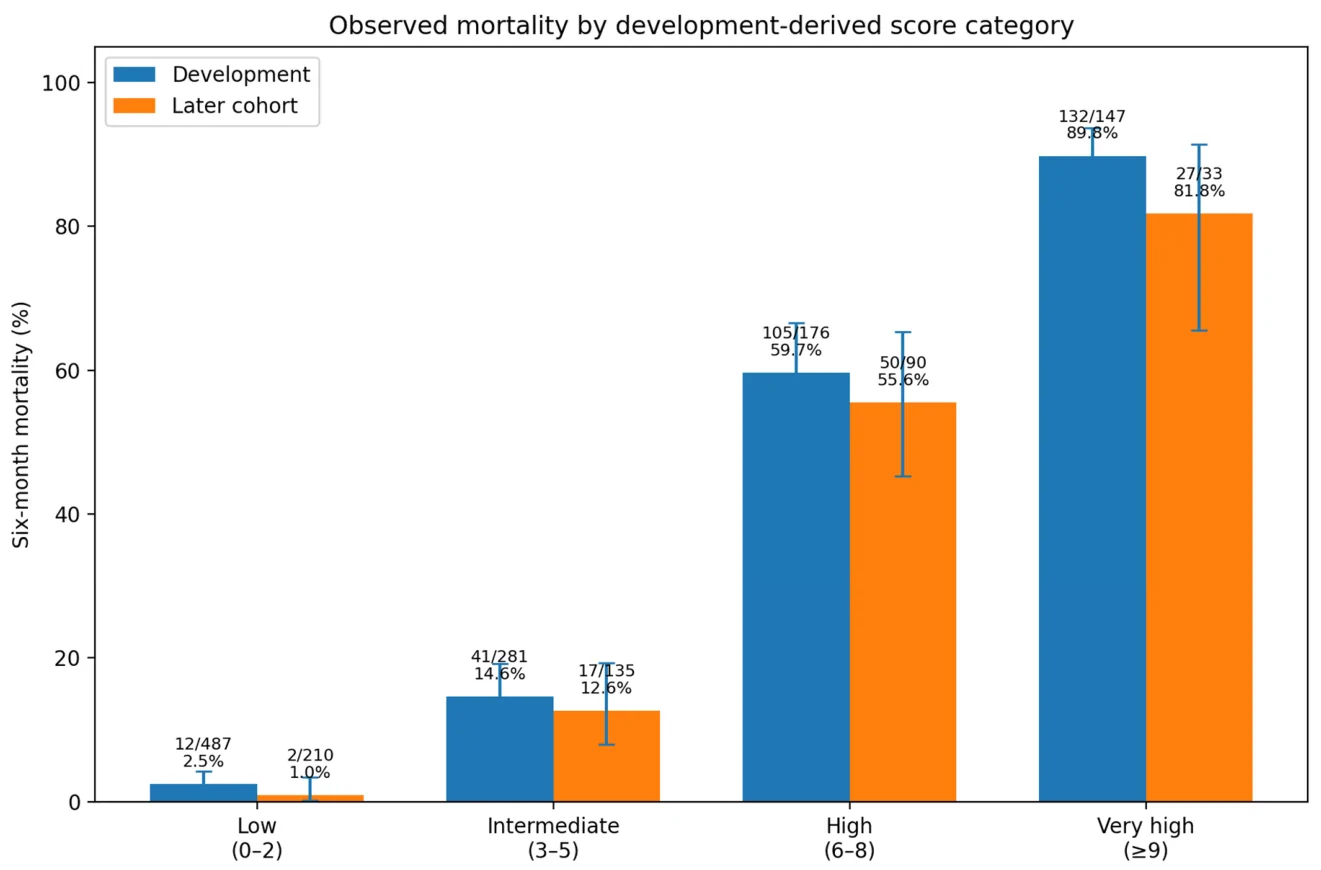
Observed six-month mortality by the development-derived simplified score category in the chronological development and later cohorts, with Wilson 95% confidence intervals and exact event counts.

The simplified score was derived exclusively in the chronological development cohort of 1091 patients and 290 deaths. Development-cohort coefficients yielded one point each for age ≥ 70 years, albumin < 3.5 g/dL, BUN ≥ 30 mg/dL and LDH > 250 U/L; two points for NLR ≥ 10; and three points each for troponin I > 34 ng/L and sodium ≥ 145 mmol/L. The fixed score had an AUC of 0.927 (95% CI 0.909–0.943) in the development cohort and 0.916 (95% CI 0.885–0.944) when applied unchanged to the later cohort. Observed mortality increased from 2.5% to 89.8% across score categories in the development cohort and from 1.0% to 81.8% in the later cohort. Two of 210 later-cohort patients classified as low risk died, precluding use of the score alone to rule out adverse outcome or determine discharge suitability (Table 5, Table 6 and Table 7 and Figure 5; Supplementary Tables S8 and S9).

## 4. Discussion

In this single-center retrospective cohort, a seven-predictor model combining age with albumin, NLR, BUN, LDH, troponin I and sodium measured within the first 24 h of admission provided strong risk stratification for six-month all-cause mortality. Bootstrap correction, reproducible later same-center temporal assessment (AUC 0.908), calibration analysis, sodium-handling sensitivity analyses, multiple imputation, decision-curve analysis and spline checks were used to examine model performance, robustness and clinical interpretability. Overall, the results support the biological and prognostic coherence of the model, while also showing calibration drift and the need for external validation before implementation.

The principal contribution of this study is not the identification of a previously unknown biomarker, but the integration of several inexpensive and routinely available measurements into a transparent prognostic framework. Numerous COVID-19 models were published during the pandemic, yet many had methodological weaknesses, limited validation and uncertain applicability [12]. Our model was developed with a prespecified predictor set, reported both discrimination and calibration, underwent bootstrap optimism correction and was evaluated in later admissions from the same center. These features address several limitations highlighted in critical appraisals of early COVID-19 prediction research. Nevertheless, the high AUC should not be interpreted as proof of superiority over established tools. A direct comparison with the 4C Mortality Score was not possible because respiratory rate, peripheral oxygen saturation on room air or supplemental oxygen, and level of consciousness were not recorded with sufficient completeness and standardization in the retrospective dataset to permit reliable calculation of the score [13].

The six-month outcome broadens the clinical perspective beyond the index hospitalization. Of the 437 deaths in the source cohort, 60 occurred after discharge or outside the study hospital. Thus, a non-negligible proportion of adverse outcomes would have been missed if follow-up had ended at discharge. The model therefore identifies not only acute in-hospital risk but also patients with persistent vulnerability after the acute episode. This does not establish that the laboratory variables directly cause late mortality; rather, they may summarize baseline frailty, organ reserve and the cumulative physiological burden of the acute illness.

Missing data are another important consideration. Complete predictor data were available for 1562 of 1827 patients (85.5%); after exclusion of three sodium values ≥ 160 mmol/L, the primary analytic cohort comprised 1559 patients (85.3%). The 268 patients not included in primary modeling accounted for 51 six-month deaths (19.0%), compared with 386 deaths among 1559 included patients (24.8%). This difference was statistically evaluated with a chi-square test (*p* = 0.042) and suggests that analytic-cohort selection may have been associated with prognosis and may have affected absolute-risk calibration. Multiple-imputation sensitivity analysis used the sodium-outlier-excluded source cohort, generated 20 imputations and yielded concordant predictor directions with a mean AUC of 0.929, supporting the robustness of predictor directions and overall discrimination. Nevertheless, informative missingness cannot be excluded because laboratory availability may reflect clinical workflow, disease severity or admission circumstances. The model should therefore be interpreted primarily as a risk-stratification tool for patients with complete admission laboratory assessment, and residual selection bias remains possible.

The biological coherence of the predictors supports the face validity of the model. Age is a consistent determinant of adverse COVID-19 outcomes and reflects immunosenescence, reduced physiological reserve and greater comorbidity burden [1,2,3,4]. Hypoalbuminaemia has repeatedly been associated with severe disease and mortality in COVID-19 [7]. NLR integrates neutrophilia, which may reflect innate immune activation and stress responses, with lymphopenia, a common feature of severe viral illness, and has shown prognostic value across multiple cohorts [6]. The combination of age, albumin and NLR therefore captures both baseline vulnerability and the inflammation–nutrition axis.

Albumin, BUN, LDH and troponin I provide complementary information on systemic vulnerability and organ stress. Hypoalbuminaemia may reflect poor nutritional reserve, systemic inflammation, capillary leakage, hepatic synthetic dysfunction and acute-phase redistribution. BUN may rise because of impaired renal function, reduced renal perfusion, dehydration, gastrointestinal bleeding or corticosteroid-related catabolism; initial BUN has been associated with in-hospital mortality in COVID-19 [8] and is also incorporated into established COVID-19 risk scores [13]. LDH is a nonspecific marker of cellular injury and may reflect pulmonary parenchymal damage, hypoxic tissue injury and systemic disease burden; a systematic review and meta-analysis supported its prognostic value for adverse COVID-19 outcomes [9]. In our model, LDH had the largest adjusted effect among the log-transformed laboratory variables, consistent with its role as an integrated marker of disease burden. Troponin elevation identifies myocardial injury and may occur through supply–demand imbalance, inflammatory injury, microvascular thrombosis or decompensation of pre-existing cardiovascular disease. Cardiac injury has been strongly associated with mortality in hospitalized patients with COVID-19 [10]. A recent narrative review likewise identified elevated troponin and BUN as markers of severe infection and LDH as an independent risk factor for death [20]. In a contemporary cohort of 1222 hospitalized patients, admission hs-troponin T, urea, albumin and LDH were jointly included in a biomarker model for fatal outcome prediction, supporting an integrated multiorgan risk framework [21]. Together, these markers connect inflammation, malnutrition, renal/hemodynamic stress, tissue injury and cardiac injury into a biologically plausible risk profile.

Sodium deserves particular caution because an apparently strong linear coefficient can be influenced by a small number of extreme observations. Three verified first admission values at or above 160 mmol/L (160, 166 and 217 mmol/L) were therefore excluded from primary model development. The retention and winsorized models each yielded an AUC of 0.936, identical to the rounded primary-model AUC, and the categorical sodium model also yielded an AUC of 0.936. Thus, overall discrimination was not driven solely by the handling of the three extreme values. These associations should not be interpreted causally: hypernatremia may reflect dehydration, impaired renal concentrating ability, severe systemic illness, care processes or terminal physiology rather than a direct causal effect. Nevertheless, the magnitude of the sodium coefficient and the three-point score assignment for hypernatremia should be considered provisional and should not guide clinical decisions until independently validated and, if necessary, recalibrated.

The chronological validation results provide an important reality check. The later cohort retained good discrimination, with an AUC of 0.908, but the calibration slope of 0.893 indicated that predictions remained somewhat too extreme. The calibration intercept of −0.087 suggested mild overprediction of average risk in later admissions. These point estimates indicate that local recalibration may be necessary before application in later or different clinical settings. The changes may reflect differences in patient selection, disease severity, clinical experience, supportive care or treatment during the 2020 study period. More broadly, they illustrate why internal validation alone is insufficient: a model can appear stable under bootstrap resampling yet require recalibration when the underlying case mix or event rate changes [17].

### 4.1. Clinical Implications

The simplified score was created to improve interpretability at the bedside. Its thresholds, binary coefficients, integer weights and category boundaries were determined using only the chronological development cohort and were then applied unchanged to the later cohort, thereby avoiding outcome leakage from the evaluation sample. The score retained strong discrimination in the later cohort (AUC 0.916), and mortality increased monotonically from 1.0% in the low-risk group to 81.8% in the very-high-risk group. This constitutes an internal–temporal evaluation of the score rather than a descriptive full-cohort check; however, both cohorts originated from the same center, and the later very-high-risk group contained only 33 patients. The score should therefore remain a supportive risk-communication and stratification tool rather than a stand-alone clinical decision rule. Two patients in the later low-risk group died. The three-point assignment for sodium ≥ 145 mmol/L should be interpreted cautiously because the sodium effect may be sensitive to case mix, the prevalence of marked abnormalities and non-linearity at extreme values. This weight remains provisional pending external validation and potential recalibration.

Clinical utility depends on the decision that the model is intended to support. After external validation and recalibration, a high predicted risk could support closer physiological monitoring during hospitalization, earlier senior or critical-care review, prioritization for structured discharge planning, or intensified post-discharge follow-up in patients who survive the index admission. Exploratory decision-curve analysis in the present cohort showed higher net benefit for the model than treat-all and treat-none strategies across threshold probabilities from 0.05 to 0.80; however, this analysis remains exploratory because no model-linked clinical action was prespecified. Future validation studies should prospectively define candidate actions, compare the model with usual clinical assessment and established scores, and evaluate whether model-guided care changes management or outcomes.

Transportability to current practice is limited by the historical setting. The cohort was assembled between March and November 2020, during the wild-type/pre-vaccination phase of the pandemic, before Omicron-lineage variants and during a period of rapidly evolving admission criteria, hospital capacity and treatment strategies. Subsequent changes in population immunity following vaccination [22], variant-specific disease severity during Omicron-lineage circulation [23], and the introduction of effective treatments, particularly systemic corticosteroids [24,25], have altered baseline mortality and may also have changed the prognostic meaning of admission laboratory abnormalities. For example, improved inpatient management or earlier presentation in later waves could lower the baseline risk at a given laboratory profile, leading to overprediction unless the intercept is updated. Consequently, the reported intercept, coefficients and risk-group mortality estimates should not be applied directly to contemporary patients without recalibration and external validation. The present results are best viewed as evidence that routinely collected admission data can support robust risk modeling, rather than as a ready-to-deploy modern COVID-19 calculator.

### 4.2. Strengths and Limitations

The high apparent discrimination may partly reflect the selected complete-case population and the single-center case mix, despite bootstrap optimism correction and chronological assessment. Several limitations should be emphasized. First, the retrospective single-center design limits generalizability and permits residual confounding. Second, 268 eligible patients were not included in the primary analytic cohort because of missing predictor data or sodium values ≥ 160 mmol/L; their lower observed mortality raises the possibility that complete-case selection affected the estimated baseline risk and absolute-risk calibration. Third, although troponin I was routinely requested at admission, some results were unavailable, and the database could not distinguish among pre-analytical problems, sample rejection, incomplete testing and electronic-record transfer failures. Fourth, the cause of post-discharge death was unavailable, so the model predicts six-month all-cause mortality after hospitalization with COVID-19 rather than COVID-19-specific death. Because diagnosis could also be based on compatible thoracic CT findings and clinical context when RT-PCR was negative, some diagnostic misclassification cannot be excluded. Fifth, chronological split-sample validation within one center is weaker than independent external validation. Sixth, the cohort was assembled in 2020, limiting direct transportability to vaccinated populations, later variants and contemporary treatment pathways. No formal subgroup-performance, fairness or health-inequality analysis was performed; therefore, differential model performance across demographic or clinical subgroups remains unknown. Exploratory restricted cubic spline analyses were performed for age and sodium: age was compatible with a linear effect, whereas sodium showed modest non-linearity. Flexible functional forms for all remaining continuous predictors were not comprehensively evaluated, and residual non-linearity may still affect calibration at extreme values. Treatment variables were not incorporated because the model was designed to use information available at admission; however, evolving COVID-19 management during 2020 may have contributed to the observed temporal calibration drift. Finally, exploratory decision-curve analysis was performed retrospectively across a broad threshold range, but no model-linked clinical action or threshold range had been prospectively prespecified; therefore, the observed net benefit should not be interpreted as definitive evidence of clinical utility.

The study also has important strengths. It included a large consecutive source cohort with 437 six-month deaths and 386 events in the primary analytic sample. With seven prespecified predictor parameters, this corresponded to approximately 55 events per parameter, a favorable event-to-parameter ratio; however, contemporary guidance emphasizes that sample-size adequacy depends on more than a fixed events-per-variable rule [26]. The outcome extended beyond discharge; all predictors were anchored to the first 24 h of admission, predictor missingness and analytic-cohort selection were explicitly described, sodium-outlier sensitivity analyses were performed, internal validation addressed optimism, and chronological validation demonstrated the degree of performance loss in later patients. The provision of the model equation, an interpretable score and transparent sensitivity analyses improves reproducibility and facilitates future external validation.

## 5. Conclusions

A model combining age with six routine laboratory variables obtained within the first 24 h of admission showed strong internally validated discrimination for six-month all-cause mortality after hospitalization with COVID-19. Exclusion, retention and winsorization of three sodium values ≥ 160 mmol/L produced closely similar discrimination, supporting the robustness of the principal findings. Discrimination remained good in later admissions from the same center, but calibration drift indicated that absolute-risk estimates require local recalibration. The development-derived simplified score also retained strong discrimination when applied unchanged to later admissions, but the model and score should currently be considered supportive risk-stratification frameworks rather than deployable stand-alone clinical calculators. Independent external validation, recalibration in contemporary cohorts and prospective evaluation of clinical net benefit are required before implementation.

## Figures and Tables

**Table 1:** Derivation of the primary analytic cohort.

Cohort Stage	Patients	Six-Month Deaths	Notes
Source cohort	1827	437	All eligible hospitalized adults
Complete data for seven predictors	1562	388	265 had at least one missing predictor
Sodium ≥ 160 mmol/L	3	2	Excluded from primary modeling
Primary analytic cohort	1559	386	Complete cases after sodium exclusion
Survivors in primary cohort	1173	0	Alive at six months
Not included in primary modeling	268	51	Missing predictor data or sodium exclusion

**Table 2:** Baseline characteristics of the primary analytic cohort according to six-month survival.

Characteristic	Survivors (*n* = 1173)	Non-Survivors (*n* = 386)	*p*	Total (*n* = 1559)
Age, years, mean ± SD	62.9 ± 14.7	74.8 ± 10.9	<0.001	65.8 ± 14.8
Male sex	597 (50.9%)	233 (60.4%)	0.001	830 (53.2%)
At least one comorbidity	843 (71.9%)	324 (83.9%)	<0.001	1167 (74.9%)
Hypertension	611 (52.1%)	245 (63.5%)	<0.001	856 (54.9%)
Coronary artery disease	291 (24.8%)	116 (30.1%)	0.042	407 (26.1%)
Congestive heart failure	70 (6.0%)	46 (11.9%)	<0.001	116 (7.4%)
COPD	182 (15.5%)	86 (22.3%)	0.002	268 (17.2%)
Chronic kidney disease	40 (3.4%)	20 (5.2%)	0.117	60 (3.8%)
Diabetes mellitus	364 (31.0%)	116 (30.1%)	0.718	480 (30.8%)
Atrial fibrillation	83 (7.1%)	60 (15.5%)	<0.001	143 (9.2%)
Thoracic CT involvement	1075 (91.6%)	354 (91.7%)	0.968	1429 (91.7%)
RT-PCR positive	827 (70.5%)	279 (72.3%)	0.505	1106 (70.9%)
Hospital stay, days, median (IQR)	11 (7–15)	11 (7–17)	0.236	11 (7–15)
Tocilizumab treatment	63 (5.4%)	27 (7.0%)	0.235	90 (5.8%)
Pulse corticosteroid treatment	25 (2.1%)	28 (7.3%)	<0.001	53 (3.4%)

**Table 3:** Univariable and multivariable logistic regression for six-month all-cause mortality.

Predictor	Crude OR (95% CI)	*p*	Adjusted OR (95% CI)	*p*
Age (per year)	1.08 (1.07–1.09)	<0.001	1.05 (1.03–1.07)	<0.001
Albumin (per g/dL)	0.05 (0.03–0.07)	<0.001	0.24 (0.15–0.38)	<0.001
NLR (per doubling)	3.32 (2.88–3.83)	<0.001	1.36 (1.16–1.60)	<0.001
BUN (per doubling)	2.88 (2.47–3.35)	<0.001	1.63 (1.30–2.03)	<0.001
LDH (per doubling)	16.02 (11.74–21.87)	<0.001	4.23 (2.81–6.38)	<0.001
Troponin I (per doubling of 1 + x)	1.42 (1.36–1.47)	<0.001	1.17 (1.11–1.23)	<0.001
Sodium (per mmol/L)	1.30 (1.25–1.34)	<0.001	1.12 (1.08–1.17)	<0.001

**Table 4:** Discriminative performance of individual markers.

Marker	AUC	Youden Cut-Off	Sensitivity	Specificity	PPV	NPV
LDH	0.838	>426.8	70.7%	84.0%	59.2%	89.7%
NLR	0.836	>9.5	72.3%	82.6%	57.8%	90.1%
Albumin	0.831	≤3.3	80.3%	73.3%	49.8%	91.9%
Troponin I	0.805	>38.2	73.1%	83.8%	59.7%	90.4%
BUN	0.731	>31.3	63.2%	77.1%	47.6%	86.4%
Sodium	0.780	≥139	68.7%	77.2%	49.8%	88.2%

Note: The Youden cut-offs in Table 4 are descriptive, data-derived summaries of individual-marker performance and should not be interpreted as recommended clinical decision thresholds. Because sodium was reported in whole mmol/L increments, its operational Youden threshold is shown as ≥139 mmol/L.

**Table 5:** Apparent performance, bootstrap internal validation and single-center temporal validation of the sodium-outlier-excluded primary model.

Metric	Value
Primary analytic patients/events	1559/386
Apparent AUC (95% CI)	0.936 (0.919–0.954)
Optimism-corrected AUC	0.933
Brier score	0.079
Chronological development cohort	1091/290 events
Later same-center temporal cohort	468/96 events
Same-center temporal AUC (95% CI)	0.908 (0.871–0.939)
Same-center temporal calibration slope	0.893
Same-center temporal calibration intercept	−0.087
Same-center temporal Brier score	0.087
Simplified score development-cohort AUC (95% CI)	0.927 (0.909–0.943)
Simplified score later-cohort AUC (95% CI)	0.916 (0.885–0.944)

**Table 6:** Development-cohort-derived simplified clinical-threshold score.

Predictor	Threshold	Points
Age	≥70 years	1
Albumin	<3.5 g/dL	1
NLR	≥10	2
BUN	≥30 mg/dL	1
LDH	>250 U/L	1
Troponin I	>34 ng/L	3
Sodium	≥145 mmol/L	3

Note: Points were derived exclusively from binary-predictor coefficients estimated in the chronological development cohort. The three-point weight assigned to sodium ≥145 mmol/L remains provisional and should not be used for clinical decisions until independently validated in an external cohort and, if necessary, recalibrated.

**Table 7:** Observed six-month mortality by the development-derived simplified score category in the development and later cohorts.

Risk Group	Score	*n*	Deaths	Mortality	95% CI
Dev: Low	0–2	487	12	2.5%	1.4–4.3%
Dev: Intermediate	3–5	281	41	14.6%	10.9–19.2%
Dev: High	6–8	176	105	59.7%	52.3–66.6%
Dev: Very high	≥9	147	132	89.8%	83.8–93.7%
Later: Low	0–2	210	2	1.0%	0.3–3.4%
Later: Intermediate	3–5	135	17	12.6%	8.0–19.2%
Later: High	6–8	90	50	55.6%	45.3–65.4%
Later: Very high	≥9	33	27	81.8%	65.6–91.4%

## Data Availability

The dataset cannot be made publicly available because it contains potentially identifiable clinical information obtained from hospital records and Türkiye’s e-Nabız national electronic health record system. Access to a de-identified dataset may be granted by the corresponding author, Onur Çelik (doktoronurcelik@yahoo.com), upon reasonable request, subject to institutional and ethical approval and applicable data-protection requirements.

## Supplementary Materials

The following supporting information can be downloaded at: https://www.mdpi.com/article/10.3390/medicina62081478/s1, Supplementary Table S1: TRIPOD+AI reporting checklist; Supplementary Methods S1: Final full-primary-cohort model specification and worked example; Supplementary Table S2: Predictor definitions, units and model handling; Supplementary Table S3: Chronological split of the sodium-outlier-excluded primary analytic cohort (*n* = 1559); Supplementary Table S4: Sodium sensitivity-analysis specifications and summary findings; Supplementary Methods S2: Reproducible analysis workflow; Supplementary Methods S3: Multiple-imputation sensitivity analysis; Supplementary Methods S4: Decision-curve analysis; Supplementary Note S1: Intended use and unavailable inputs; Supplementary Note S2: Protocol, registration, and patient involvement; Supplementary Figure S1: Participant-flow diagram; Supplementary Table S5: Multiple-imputation pooled sensitivity results; Supplementary Figure S2: Decision-curve analysis; Supplementary Table S6: Selected net-benefit values from decision-curve analysis; Supplementary Methods S5: Restricted cubic spline assessment; Supplementary Table S7: Restricted cubic spline assessment for age and sodium; Supplementary Figure S3: Restricted cubic spline assessment for age; Supplementary Figure S4: Restricted cubic spline assessment for sodium; Supplementary Methods S6: Development-cohort derivation and internal-temporal evaluation of the simplified score; Supplementary Table S8: Development-cohort binary-predictor coefficients and assigned score weights; Supplementary Table S9: Development and later-cohort performance of the development-derived simplified score.
