# Supplementary material for: Admission Laboratory-Based Prediction of Six-Month Mortality After COVID-19 Hospitalization: Model Development, Bootstrap Internal Validation, and Single-Center Temporal Validation
Source: Medicina (Kaunas). 2026 Jul 31;62(8):1478. doi: 10.3390/medicina62081478 (PMC13515301; doi:10.3390/medicina62081478)
Supplement: Supplementary file 1 [file medicina-62-01478-s001.zip › medicina-4437444-supplementary.pdf]

Supplementary Material

Admission Laboratory-Based Prediction of Six-Month Mortality After COVID-19 Hospitalization: Model Development, Bootstrap Internal Validation, and Single-Center Temporal Validation

Includes the TRIPOD+AI checklist, participant-flow diagram, final full-primary-cohort model specification, predictor definitions, chronological partition summary, numeric sodium sensitivity analyses, reproducible workflow, multiple-imputation sensitivity analysis, decision-curve analysis, restricted cubic spline assessment and development-cohort simplified-score derivation and later-cohort evaluation based on the 1827-patient source cohort and 1559-patient primary analytic cohort.

Supplementary Table S1. TRIPOD+AI reporting checklist

This author-prepared checklist follows the official 27-item TRIPOD+AI framework for prediction-model development and evaluation. Item descriptions are summarized, and locations refer to the submitted manuscript or this supplementary file.

Item	Topic	Reporting summary	Location
1	Title	Study type, population, outcome, bootstrap internal validation and same-center temporal validation identified	Title
2	Abstract	Structured summary of data source, model, validation, performance and intended use	Abstract
3a	Background	Clinical context, rationale and existing models described	Introduction
3b	Intended use	Target population, intended users and supportive risk-stratification role described	Introduction; Discussion 4.1; Supplementary Note S1
3c	Health inequalities	No formal fairness or inequality analysis was performed; this limitation is stated	Discussion 4.2
4	Objectives	Development, bootstrap internal validation, same-center temporal assessment and score derivation specified	End of Introduction
5a	Data source	Consecutive retrospective hospital cohort and rationale described	Methods 2.1
5b	Dates	Accrual period and six-month outcome horizon reported	Methods 2.1–2.2
6a	Setting	Single tertiary referral center in Türkiye	Methods 2.1
6b	Eligibility	Adult hospitalized COVID-19 eligibility described	Methods 2.1
6c	Treatments	Post-admission treatments excluded from the admission model; temporal treatment drift discussed	Methods 2.3; Discussion 4.2
7	Data preparation	First-24-hour measurements, transformations and sodium quality review reported	Methods 2.3–2.5; Supplementary Table S2
8a	Outcome	Six-month all-cause mortality, ascertainment and time horizon defined	Methods 2.2
8b	Outcome assessors	Hospital records and national electronic health record vital status used	Methods 2.2
8c	Outcome blinding	Not applicable to routinely recorded vital status	Methods 2.2
9a	Predictor choice	Seven predictors selected a priori; no automated selection	Methods 2.3
9b	Predictor definitions	Timing, units, assay and model handling reported	Methods 2.3; Supplementary Table S2
9c	Predictor assessors	Objective laboratory measurements obtained through routine clinical systems	Methods 2.3
10	Study size	All consecutive eligible patients used; 1559 patients and 386 events in the primary model; approximately 55 events per predictor parameter	Methods 2.1; Results 3.1; Discussion 4.2
11	Missing data	Complete-case analysis and a 20-imputation chained-equation sensitivity analysis reported	Methods 2.4; Results 3.4; Discussion 4.2; Supp. Methods S3
12a	Data use/partition	500-bootstrap internal validation and chronological 70%/30% split of the 1559-patient primary analytic cohort described	Methods 2.5
12b	Predictor handling	Original scales and $\log_2$ transformations specified	Methods 2.5; Supplementary Table S2
12c	Model building	Logistic regression with fixed prespecified predictor set	Methods 2.5
12d	Clustering	Not applicable; single-center cohort	Methods 2.1
12e	Performance	AUC, calibration intercept/slope, LOESS calibration and Brier score reported	Methods 2.5; Results 3.4; Table 5
12f	Updating	No model updating performed; recalibration need discussed	Discussion; Conclusions

Item	Topic	Reporting summary	Location
12g	Prediction calculation	Full equation and worked example supplied	Results 3.3; Supplementary Methods S1
13	Class imbalance	No outcome resampling or class-imbalance correction used	Methods 2.5
14	Fairness	No formal fairness adjustment or subgroup performance analysis performed	Discussion 4.2
15	Model output	Individual probabilities and a 0–12 integer score derived exclusively in the chronological development cohort and applied unchanged to the later cohort	Methods 2.5; Results 3.5; Tables 5–7; Supp. Methods S6; Tables S8-S9
16	Development vs evaluation	Chronological development/evaluation separation reported for both the continuous model and simplified score	Methods 2.5; Supplementary Tables S3 and S9
17	Ethics	Committee approval and consent waiver reported	Methods 2.1; Institutional Review Board Statement
18a	Funding	No external funding	Funding
18b	Conflicts	No conflicts declared	Conflicts of Interest
18c	Protocol	No prospective protocol prepared	Supplementary Note S2
18d	Registration	Study not registered	Supplementary Note S2
18e	Data sharing	Availability subject to ethical and institutional approval	Data Availability Statement
18f	Code sharing	No separate analysis-code file accompanies the submission; the reproducible workflow and full model specification are described in detail.	Supplementary Methods S1-S2
19	Patient/public involvement	No patient or public involvement	Supplementary Note S2
20a	Participant flow	Source, complete-case and final analytic counts reported	Results 3.1; Table 1
20b	Characteristics	Baseline characteristics, events and aggregate missingness reported	Tables 1–2
20c	Evaluation comparison	Development and later-cohort sizes, event rates, continuous-model performance and development-derived score performance summarized	Supplementary Tables S3 and S9
21	Analysis counts	Participants and events reported for primary and temporal analyses	Table 5; Supplementary Table S3
22	Full model	Final full-primary-cohort intercept, coefficients, transformations and probability formula supplied; distinction from chronological development model stated	Results 3.3; Supplementary Methods S1
23a	Performance	Point estimates and confidence intervals reported; calibration and ROC figures supplied in main manuscript	Results 3.4; Table 5; Figures 1, 3 and 4
23b	Cluster heterogeneity	Not applicable; single center	Methods 2.1
24	Model updating	No updating performed	Not applicable
25	Interpretation	Results interpreted against prior models and clinical context	Discussion
26	Limitations	Missingness, possible selection effects, nonlinearity, historical setting and validation limits discussed	Discussion 4.2
27a	Unavailable inputs	Model intended for patients with complete first-24-hour predictor measurements	Discussion 4.1–4.2; Supplementary Note S1
27b	User interaction	Full equation requires software; integer score can be calculated manually	Supplementary Methods S1; Supplementary Note S1
27c	Next steps	External validation, contemporary recalibration and prospective clinical-utility studies proposed	Discussion; Conclusions

Supplementary Methods S1. Final full-primary-cohort model specification and worked example

The equation below is the final model estimated in the full sodium-outlier-excluded primary analytic cohort (n=1559) for reporting and future external validation. It is distinct from the coefficient set estimated in the earliest 70% of admissions and applied unchanged to the latest 30% for the same-center chronological assessment. Age, albumin and sodium are entered in their natural clinical units; NLR, BUN and LDH are $\log_2$-transformed; and troponin I is transformed as $\log_2(1 + \text{troponin I})$.

$$\text{logit}(p) = -32.7703 + 0.05021 \times \text{age} - 1.42211 \times \text{albumin}$$
$$+ 0.31019 \times \log_2(\text{NLR}) + 0.48726 \times \log_2(\text{BUN})$$

```
          + 1.44255 × log₂(LDH) + 0.15598 × log₂(1 + troponin I)
          + 0.11751 × sodium

  p = exp[logit(p)] / {1 + exp[logit(p)]}
```

Worked example: for age 75 years, albumin 3.0 g/dL, NLR 12, BUN 40 mg/dL, LDH 500 U/L, troponin I 50 ng/L and sodium 142 mmol/L, the linear predictor is approximately 0.939 and the estimated six-month mortality probability is approximately 71.9%. This example illustrates calculation only and is not a treatment, ICU-admission or discharge recommendation.

Supplementary Table S2. Predictor definitions, units and model handling

Predictor	Unit	Definition	Model handling	Timing/quality rule
Age	Years	Chronological age at index admission	Original scale	First admission record
Albumin	g/dL	Serum albumin	Original scale	First valid value within 24 h
NLR	Ratio	Absolute neutrophil count divided by absolute lymphocyte count	$\log_2$	Calculated from first complete blood count within 24 h
BUN	mg/dL	Blood urea nitrogen	$\log_2$	First valid value within 24 h
LDH	U/L	Lactate dehydrogenase	$\log_2$	First valid value within 24 h
Troponin I	ng/L	Cardiac troponin I; paramagnetic-particle chemiluminescent immunoassay, UniCel DxI 800 Access Immunoassay System (Beckman Coulter, Inc., Brea, CA, USA)	$\log_2(1 + x)$	Routinely requested; first retrievable value within 24 h; local 99th-percentile upper reference limit 34 ng/L
Sodium	mmol/L	Serum sodium	Original scale	First valid value within 24 h; values ≥160 mmol/L excluded from primary model

Supplementary Table S3. Chronological split of the sodium-outlier-excluded primary analytic cohort (n=1559)

Patients in the primary analytic cohort were ordered by admission date. Coefficients estimated in the earliest 70% (1091 patients; 290 deaths) were applied unchanged to the latest 30% (468 patients; 96 deaths). The 70%/30% split fell within admissions dated 5 October 2020; records sharing this date were retained in their original source-row order as a reproducible tie-breaking rule. Because both partitions originated from the same institution, this assessment is internal-temporal rather than external validation. The final full-cohort equation reported in Supplementary Methods S1 was estimated separately in all 1559 patients and was not used to generate the later-cohort performance estimates.

Metric	Development: earliest 70% of primary cohort	Later same-center cohort: latest 30% of primary cohort
Patients / events	1091 / 290	468 / 96
Six-month mortality	290/1091 (26.6%)	96/468 (20.5%)
Analytic role	Coefficient estimation	Unchanged model application
AUC (95% CI)	Not reported as a validation estimate	0.908 (0.871–0.939)
Calibration slope	Not applicable	0.893
Calibration intercept	Not applicable	−0.087
Brier score	Not reported as a validation estimate	0.087
Analytic source	Sodium-outlier-excluded primary analytic cohort (n=1559)	Sodium-outlier-excluded primary analytic cohort (n=1559)

Supplementary Table S4. Sodium sensitivity-analysis specifications and summary findings

During final data verification, three first valid admission sodium results were confirmed as 160, 166 and 217 mmol/L. The primary analysis excluded values ≥160 mmol/L. Alternative analyses assessed whether the main findings were dependent on this data-quality decision. Sodium associations should not be interpreted causally, and the three-point score weight for sodium ≥145 mmol/L remains provisional pending independent external validation and possible recalibration.

Analysis	n / events	Sodium handling	Verified numeric result
Primary analysis	1559 / 386	Exclude sodium ≥160 mmol/L	Adjusted sodium OR 1.12 per mmol/L (95% CI 1.08–1.17); AUC 0.936 (95% CI 0.919–0.954).
Retention analysis	1562 / 388	Retain all three values ≥160 mmol/L	Adjusted sodium OR 1.09 per mmol/L (95% CI 1.06–1.13); AUC 0.936.
Winsorized analysis	1562 / 388	Set sodium values >160 mmol/L to 160 mmol/L	Adjusted sodium OR 1.12 per mmol/L (95% CI 1.08–1.16); AUC 0.936.
Categorical sensitivity model	1559 / 386	Model sodium as <135, 135–144 and ≥145 mmol/L	Sodium ≥145 vs <135 mmol/L: OR 9.18 (95% CI 4.38–19.24); AUC 0.936.

Note: All sodium-effect estimates and AUC values shown above are from the verified analysis of the current 1827-patient source cohort using first valid admission sodium results. The nearly identical AUCs indicate that overall discrimination was insensitive to exclusion, retention, winsorization or categorical handling of the three extreme sodium values. The categorical comparison (sodium ≥145 versus <135 mmol/L) was specified within the sensitivity model and should be interpreted as a robustness analysis rather than as a clinical threshold effect estimate.

Supplementary Methods S2. Reproducible analysis workflow

Statistical analyses were performed using IBM SPSS Statistics version 32.0.0.0 (IBM Corp., Armonk, NY, USA) and Python version 3.14.6 (Python Software Foundation, Wilmington, DE, USA).

Step	Procedure
1	Convert admission-date serial values to calendar dates and define six-month mortality as TOTAL MORT. > 0.
2	Calculate NLR from absolute neutrophil and lymphocyte counts.
3	Retain patients with complete values for the seven prespecified predictors; exclude sodium ≥160 mmol/L in the primary analysis.
4	Apply $\log_2$ transformations to NLR, BUN and LDH and $\log_2(1 + x)$ to troponin I.
5	Fit a seven-predictor logistic regression without automated predictor selection.
6	Quantify apparent AUC, calibration intercept and slope, LOESS calibration, and Brier score; estimate AUC optimism using 500 bootstrap resamples.
7	Order patients by admission date; fit the model in the earliest 70% and apply coefficients unchanged to the latest 30%.
8	Repeat relevant analyses under alternative sodium handling and conduct a 20-imputation chained-equation sensitivity analysis.
9	Fit the seven binary score components and derive integer weights exclusively in the chronological development cohort; fix the point structure and risk-group boundaries; then apply them unchanged to the later cohort.

Supplementary Methods S3. Multiple-imputation sensitivity analysis

Twenty chained-equation imputations were generated in the sodium-outlier-excluded source cohort (n=1824; 435 deaths) to assess the influence of missing predictor values. Rubin's rules were used to pool coefficient estimates and standard errors. Predictor directions were concordant with the complete-case model: age, NLR, BUN, LDH, troponin I and sodium remained positively associated with six-month mortality, whereas albumin remained inversely associated. Mean MI AUC was 0.929 (SD 0.001; range 0.927–0.931). Because the reasons for missing laboratory measurements were not reliably documented, the plausibility of the missing-at-random assumption underlying the imputation analysis could not be adequately assessed. The analysis therefore supports the robustness of predictor directions and overall discrimination but does not replace the complete-case primary model, which is intended for patients with all required admission predictors available. Pooled MI results are summarized in Supplementary Table S5.

Supplementary Methods S4. Decision-curve analysis

Decision-curve analysis was performed as an exploratory assessment of clinical utility. Net benefit was calculated across threshold probabilities from 0.05 to 0.80 and compared with default treat-all and treat-none strategies. The prediction model showed higher net benefit than both default strategies across this range in the primary cohort. Because no model-linked clinical action was prospectively specified, these findings should be interpreted as supportive rather than definitive evidence of clinical utility. The decision curve is shown in Supplementary Figure S2, and selected net-benefit values are summarized in Supplementary Table S6. Net benefit was calculated according to the method of Vickers and Elkin. At each of the 76 evaluated thresholds (0.05 to 0.80 in increments of 0.01), model net benefit exceeded both treat-all and treat-none net benefit.

Supplementary Note S1. Intended use and unavailable inputs

The model is intended for supportive risk stratification in adults hospitalized with COVID-19 who have complete first-24-hour predictor measurements. The full equation requires an electronic calculator or software implementation; the integer score can be calculated manually by healthcare professionals. Neither tool should be used as a stand-alone discharge, ICU-admission or treatment-escalation rule. If a required predictor is unavailable, the complete-case model and simplified score should not be calculated by substituting an assumed normal value.

Supplementary Note S2. Protocol, registration, and patient involvement

No prospective study protocol was prepared, the study was not registered, and patients or members of the public were not involved in the design, conduct, analysis, reporting or dissemination planning of this retrospective study.

Supplementary Figure S1. Participant-flow diagram

The flow diagram summarizes the source cohort, exclusions for missing predictors, sodium quality-control exclusion and final primary analytic cohort.

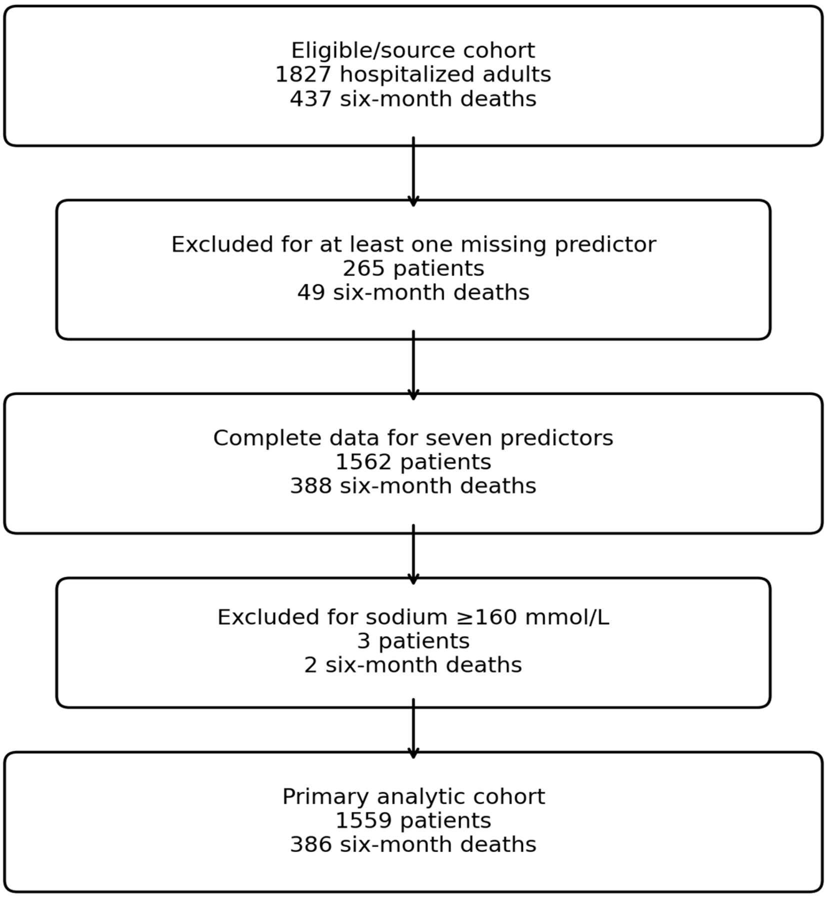

Supplementary Table S5. Multiple-imputation pooled sensitivity results

The table summarizes pooled logistic-regression estimates across 20 chained-equation imputations in the sodium-outlier-excluded source cohort (n=1824; 435 deaths).

Predictor	Pooled beta	Pooled OR (95% CI)
Age (per year)	0.049	1.05 (1.04–1.07)
Albumin (per g/dL)	−1.362	0.26 (0.17–0.39)
$\log_2$(NLR)	0.355	1.43 (1.22–1.67)
$\log_2$(BUN)	0.221	1.25 (0.94–1.66)
$\log_2$(LDH)	1.323	3.75 (2.60–5.42)
$\log_2$(1 + troponin I)	0.145	1.16 (1.11–1.21)
Sodium (per mmol/L)	0.121	1.13 (1.09–1.17)

Mean MI AUC was 0.929 (SD 0.001; range 0.927–0.931).

Supplementary Figure S2. Decision-curve analysis

Exploratory decision-curve analysis compared the prediction model with treat-all and treat-none strategies across threshold probabilities from 0.05 to 0.80.

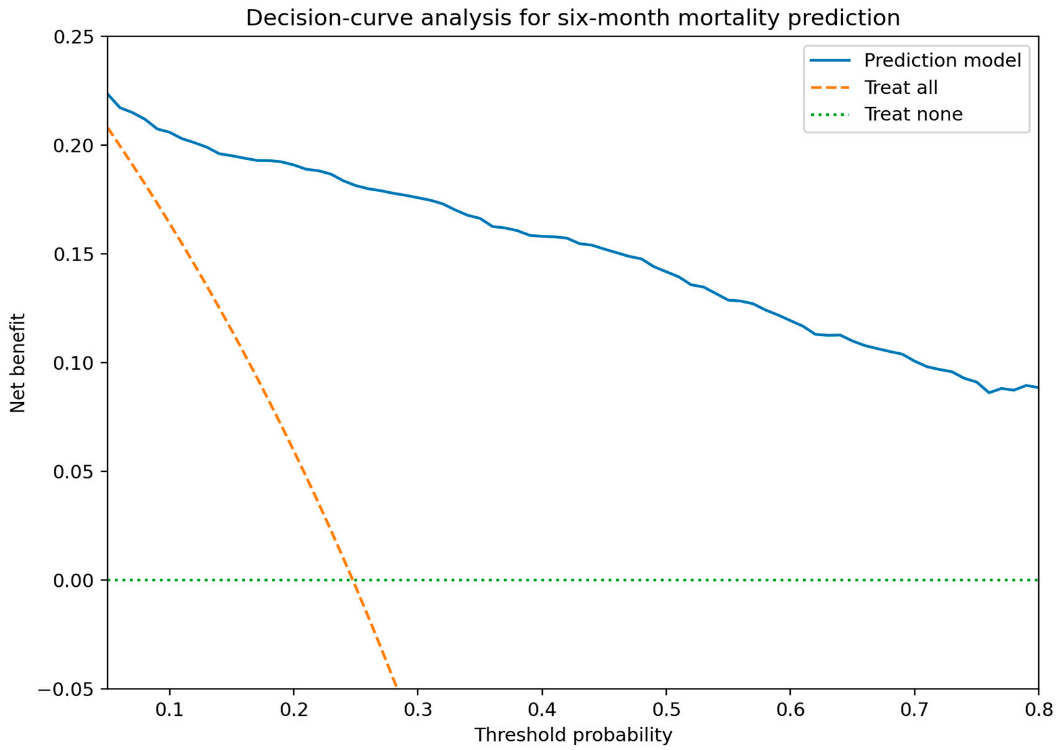

Supplementary Table S6. Selected net-benefit values from decision-curve analysis

Threshold probability	Model net benefit	Treat-all net benefit	Treat-none net benefit
0.05	0.223	0.208	0.000
0.10	0.206	0.164	0.000
0.20	0.191	0.059	0.000
0.30	0.176	−0.075	0.000
0.40	0.158	−0.254	0.000
0.50	0.142	−0.505	0.000
0.60	0.119	−0.881	0.000
0.70	0.101	−1.508	0.000
0.80	0.089	−2.762	0.000

Supplementary Methods S5. Restricted cubic spline assessment

Restricted cubic spline sensitivity analyses using four knots—two internal knots and two boundary knots—were performed for age and sodium. Non-linearity was assessed by likelihood-ratio tests comparing the spline and linear specifications in otherwise identical seven-predictor logistic models; each test had two degrees of freedom. These analyses were exploratory and intended to assess model adequacy rather than to refit the primary parsimonious model. The four knot locations were 18.00, 44.67, 71.33 and 99.00 years for age and 116.00, 133.33, 145.67 and 159.00 mmol/L for sodium. Each spline model retained the other six predictors with the same transformations as the primary model. For graphical predictions, the remaining predictors were held at their cohort median values. Predicted probabilities rather than relative odds were displayed, so no single exposure reference value was required.

Supplementary Table S7. Restricted cubic spline assessment for age and sodium

Spline specification note: the listed knot values comprise the lower boundary, two internal knots and upper boundary, respectively. The non-linearity p values refer to likelihood-ratio comparisons with the corresponding linear term in an otherwise identical seven-predictor model.

Variable	Number of knots	Non-linearity test df	Likelihood-ratio statistic	p for non-linearity
Age	4	2	3.269	0.195
Sodium	4	2	8.362	0.015

The age association did not show evidence of important non-linearity (p=0.195). Sodium showed modest non-linearity (p=0.015), supporting cautious interpretation of its linear coefficient and point-score weight pending external validation and recalibration.

Supplementary Figure S3. Restricted cubic spline assessment for age

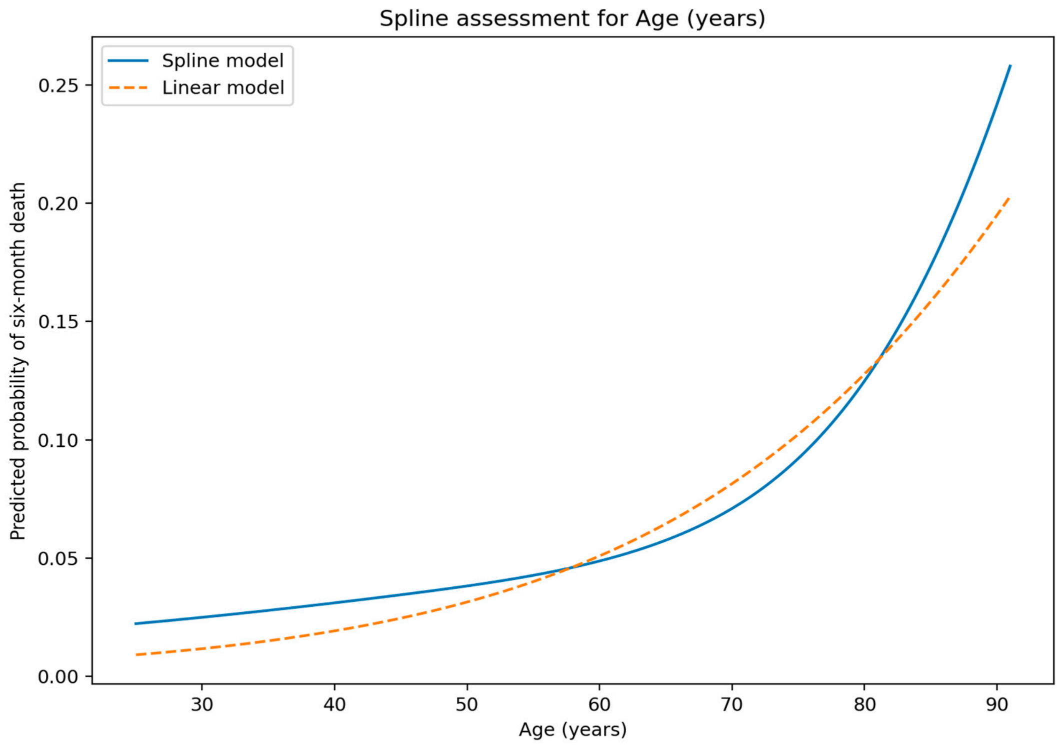

Supplementary Figure S4. Restricted cubic spline assessment for sodium

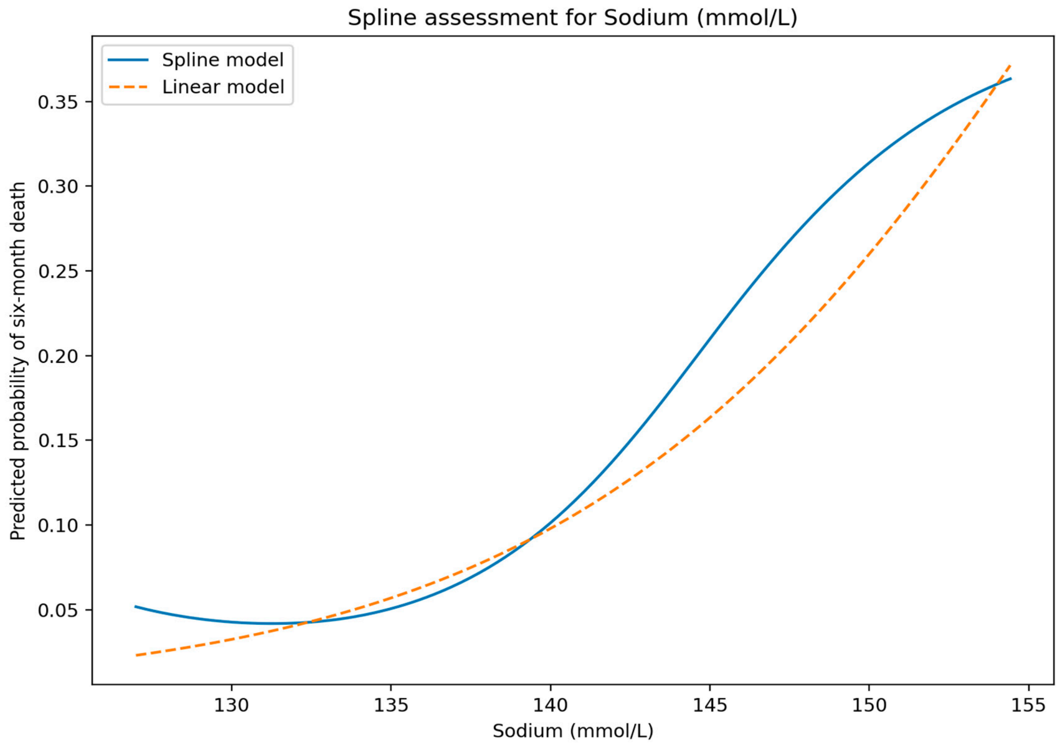

Supplementary Methods S6. Development-cohort derivation and internal-temporal evaluation of the simplified score

The simplified score was developed exclusively in the chronological development cohort (1091 patients; 290 deaths). Seven clinically interpretable binary thresholds were fixed before score fitting and were not re-estimated in the later cohort: age ≥70 years, albumin <3.5 g/dL, NLR ≥10, BUN ≥30 mg/dL, LDH >250 U/L, troponin I >34 ng/L and sodium ≥145 mmol/L. A logistic regression containing all seven binary predictors was fitted in the development cohort. For a transparent and reproducible three-tier integerization, beta coefficients <1.25 were assigned one point, coefficients from 1.25 to <1.65 were assigned two points, and coefficients ≥1.65 were assigned three points. This yielded a 0–12 score. Risk-group boundaries were fixed as low (0–2), intermediate (3–5), high (6–8) and very high (≥9) before the score was applied to the later cohort. No threshold, coefficient band, point weight or risk-group boundary was modified after examination of later-cohort outcomes. Development and later-cohort AUCs were accompanied by 95% percentile-bootstrap confidence intervals from 5000 resamples. Observed mortality within score categories was summarized with Wilson 95% confidence intervals.

Supplementary Table S8. Development-cohort binary-predictor coefficients and assigned score weights

Binary component	Development-cohort beta	Development-cohort OR	Assigned points
Age ≥70 years	0.960	2.61	1
Albumin <3.5 g/dL	1.036	2.82	1
NLR ≥10	1.572	4.82	2
BUN ≥30 mg/dL	1.098	3.00	1
LDH >250 U/L	1.071	2.92	1
Troponin I >34 ng/L	1.678	5.36	3
Sodium ≥145 mmol/L	2.267	9.65	3

Only development-cohort outcomes were used to estimate the binary coefficients and assign points. The score structure was locked before later-cohort evaluation. Sodium and troponin I received the largest weights because their development-cohort coefficients exceeded 1.65. The sodium weight remains provisional pending independent external validation.

Supplementary Table S9. Development and later-cohort performance of the development-derived simplified score

Cohort	AUC (95% CI)	Risk group (score)	n / deaths	Mortality (95% CI)
Development	0.927 (0.909–0.943)	Low (0–2)	487 / 12	2.5% (1.4–4.3%)
Development		Intermediate (3–5)	281 / 41	14.6% (10.9–19.2%)
Development		High (6–8)	176 / 105	59.7% (52.3–66.6%)
Development		Very high (≥9)	147 / 132	89.8% (83.8–93.7%)
Later	0.916 (0.885–0.944)	Low (0–2)	210 / 2	1.0% (0.3–3.4%)
Later		Intermediate (3–5)	135 / 17	12.6% (8.0–19.2%)
Later		High (6–8)	90 / 50	55.6% (45.3–65.4%)
Later		Very high (≥9)	33 / 27	81.8% (65.6–91.4%)

The point structure, score categories and all score-development decisions were fixed using only the development cohort and were applied unchanged to the later cohort. The later-cohort results therefore represent an internal-temporal evaluation of the simplified score, not external validation.

Checklist reference: Collins GS, Moons KGM, Dhiman P, et al. TRIPOD+AI statement: updated guidance for reporting clinical prediction models that use regression or machine learning methods. BMJ. 2024;385:e078378. doi:10.1136/bmj-2023-078378.

Supplementary methodological reference: Vickers AJ, Elkin EB. Decision curve analysis: a novel method for evaluating prediction models. Med Decis Making. 2006;26(6):565–574. doi:10.1177/0272989X06295361.
